# Unified Solid Solution Product of [Nb][C] in Nb-Microalloyed Steels with Various Carbon Contents

**DOI:** 10.3390/ma17133369

**Published:** 2024-07-08

**Authors:** Yongming Yan, Yanjun Xue, Ke Liu, Wenchao Yu, Jie Shi, Maoqiu Wang

**Affiliations:** 1 Central Iron & Steel Research Institute, Co., Ltd., Beijing 100081, China; 15110562925@163.com (Y.X.); yuwenchao@nercast.com (W.Y.); shijie@nercast.com (J.S.); maoqiuwang@hotmail.com (M.W.); 2 Jianglu Machinery & Electronics Group, Co., Ltd., Xiangtan 411199, China; liuke820x@126.com

**Keywords:** Nb-microalloyed gear steel, low-pressure carburizing, NbC precipitate, solid solution product, carbon content

## Abstract

In this work, the solid solution product of [Nb][C] in the Nb-microalloyed steels with various carbon contents in the range of 0.20~1.80 wt.% was investigated by means of the extraction phase analysis method. The results showed that the Nb content in austenite tended to first decrease and then increase with the increase of carbon content in the steels. A unified solid solution product of [Nb][C] in austenite at different temperatures was obtained according to the results of the experimental steels. The Nb content in austenite of the experimental steels with high carbon contents was lower than that calculated by Ohtani’s equation. The existence of NbC precipitates in the case and the core of the specimens carburized at 930 °C and 980 °C were verified by transmission electron microscopy (TEM) observations. The pinning effect of NbC precipitates on austenite grain growth was calculated according to the size and amount of NbC precipitates in the carburized case and the core of the carburized specimens. The calculated results of prior austenite grain sizes were in good agreement with the experimental results, which indicated that the unified solid solution product of [Nb][C] in Nb-microalloyed steels with various carbon contents was applicable for the low-pressure carburizing process.

## 1. Introduction

As the key transmission components, gears are generally processed by carburizing heat treatment to improve their performance and thus withstand a sufficiently large load and contact stress [1,2]. Improving carburization efficiency by shortening carburization time is a development direction for carburization, and increasing the carburizing temperature is the most effective way to shorten the carburizing time [3,4,5]. However, the increase in carburizing temperature usually leads to abnormal grain growth in the steel, which would decrease its strength and fatigue strength [6,7]. Therefore, it is important to maintain the fine grain size of the sample after the high-temperature carburizing process, and many studies found that adding a small amount of niobium element to the steel could effectively refine the grain size by forming NbC precipitates to pin the grain boundaries in the steel [8,9,10,11].

Compared with gas carburizing, low-pressure carburizing has higher quality and is more environmentally friendly [12,13]. On the other hand, the carburizing method of low-pressure carburizing is pulse carburizing, and the surface of the specimen in the low-pressure environment will reach carbon saturation in a very short time [14,15]. This means that the carbon content on the surface of the sample during the low-pressure carburizing process is higher than that during the gas carburizing process. For example, when the carburizing temperature is 980 °C, the carbon saturation is 1.8 wt.%, and the solid solubility of Nb in austenite is 0.097 wt.% according to Ohtani’s [Nb][C] solid solubility product equation [16], which was much higher than the content of Nb usually added in Nb-microalloyed steels. This means that a large amount of NbC precipitates would dissolve during the high-temperature and low-pressure carburizing process, which cannot provide an effective pinning effect to inhibit grain growth. 

In the previous work, the extraction phase analysis method was used to study the solid solubility products of [Nb][C] in the case and the core of high-temperature carburizing steel [17]. We found that the solid solubility products of [Nb][C] in the case were lower than that calculated by Ohtani’s equation, which means that there were still enough NbC precipitates in the case to refine the grain size. However, the carbon content of the case was 1.0 wt.%, which was lower than the carbon content during the low-pressure carburizing process. Whether sufficient NbC precipitates can still be precipitated during the low-pressure carburizing process to provide sufficient pinning effect has no related research. However, we could not obtain the unified [Nb][C] solid solubility product equation used in the low-pressure carburizing process, as there were only the carbon contents of 0.2 wt.% and 1.0 wt.% considered in the previous work.

Therefore, this work was aimed to study the solid solution product of [Nb][C] in the Nb-microalloyed steels with various carbon contents in the range of 0.20~1.80 wt.%, and a unified solid solution product of [Nb][C] in Nb-microalloyed steels was expected to be obtained.

## 2. Experimental

In this work, the experimental steel was Nb-microalloyed 18CrNiMo7-6 steel, which was prepared by the electric arc furnace and ladle furnace process, and its chemical composition is shown in Table 1. Due to the decrease in carbon content from the surface to the core, it is not possible to directly use the extraction phase analysis method for the determination of solid solubility product. Therefore, experimental steels with a carbon content of 0.6 wt.%, 1.0 wt.%, 1.4 wt.%, and 1.8 wt.% were also prepared to simulate different positions during the low-pressure carburizing process in this work, and their chemical compositions are shown in Table 1. To be brief, they were named as 20C, 60C, 100C, 140C and 180C, respectively.

The specimens used for the extraction phase analysis method were machined into a diameter of 5 mm and with a length of 80 mm and then austenitized at 930 °C, 980 °C, 1050 °C, 1100 °C and 1200 °C for 3 h, with oil cooling to room temperature. After heat treatment, the extraction phase analysis method was used to extract the NbC precipitates, and the electrolyte was 10 g/L Lithium chloride solution and 10% acetylacetone methanol, with the current of 0.03 to 0.05 A/cm^2^ and the temperature of −5 to 0 °C. The contents of extracts were carried out by inductively coupled plasma-mass spectrometry (ICP-AES).

**Figure 1:**
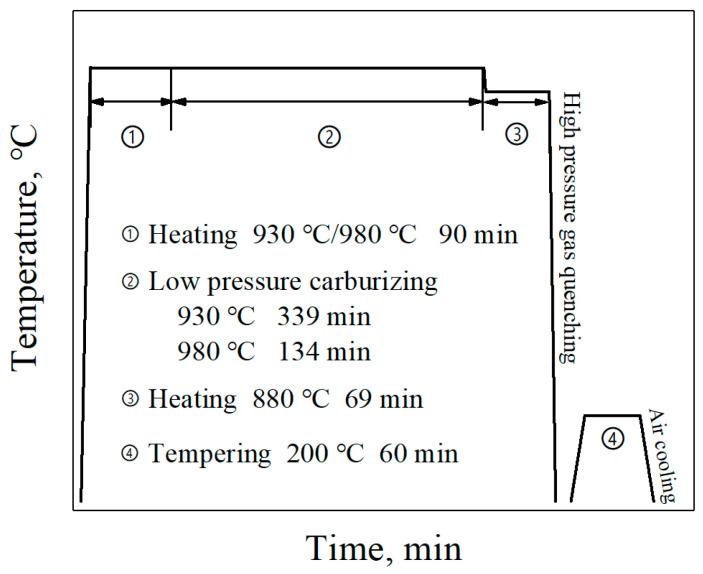
Schematic diagrams showing the low-pressure carburizing process.

A high carburizing temperature of 980 °C was used in this work, and the usual carburizing temperature of 930 °C was also conducted for comparison. The low-pressure carburizing process was carried out in the SynchroTherm type low-pressure carburizing furnace produced by ALD Vacuum Technologies GmbH (Hanau, Germany), and the detailed carburizing process was illustrated in Figure 1.

The prior austenite grains were etched in picric acid and the micrographs were observed by using the Zeiss Axio Scope A1 optical microscope produced by Carl Zeiss AG (Oberkochen, Germany). The mean prior austenite grain size of the near-surface and core of specimens was measured by the linear intercept method. The NbC precipitates were extracted by the carbon film, and the Talors F200X transmission electron microscope (TEM) produced by Thermo Fisher Scientific (Waltham, MA, USA) was used to observe the morphology of the precipitated phase, and the Super-X energy dispersive X-ray spectroscopy (EDS) produced by Thermo Fisher Scientific (Waltham, MA, USA) was used to examine the elements present.

## 3. Results and Discussion

**Figure 2:**
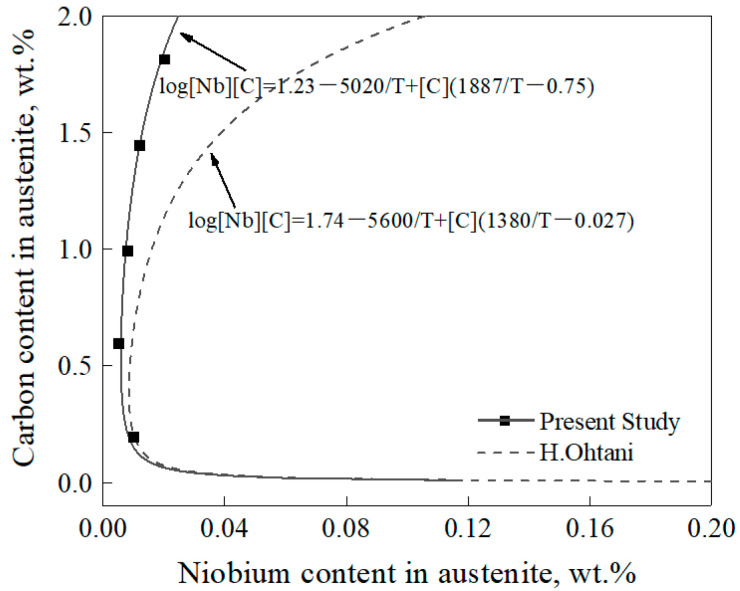
Variation of the solid solubility of Nb in austenite with carbon content at 980 °C.

The results of the solid solubility of Nb and C in the austenite and the amount of NbC precipitates with different carbon contents examined by the extraction phase analysis method are shown in Table 2. It can be seen that the solid solubility of Nb in austenite showed a trend of first decreasing and then increasing with the increase of carbon content, which was similar to the trend calculated by Ohtani’s equation, as shown in Figure 2. However, the content of Nb in austenite was lower than the result calculated by Ohtani’s equation, and the difference between the experimental results and those calculated by Ohtani’s equation was increased with the increase of carbon content. This means that the solid solubility of Nb and C in austenite was lower than the calculated result, which indicated that the NbC precipitates would not occur re-dissolution as calculated by Ohtani’s equation.

On the other hand, linear fitting was used to fit the [Nb][C] solid solubility product equation with different carbon content according to the result of extraction phase analysis, and the solid solubility product equation is expressed as [18]:

(1) $$\log\left[Nb\right]\left[C\right]=A-B/T$$

where A and B are two constants. The value of A and B of [Nb][C] solid solubility product equations in the experimental steels with different carbon content are shown in Table 3, and by fitting the relationship between the values of A and B and the carbon content to obtain the [Nb][C] solid solubility product equations under different carbon content in gear steels, which is expressed as follows:

(2) $$\log\left\{\left[Nb\right]\left[C\right]\right\}=1.23-5020/T+\left[C\right]\left(1887/T-0.75\right)$$

Comparing the results of extraction phase analysis and Ohtani’s equation, the increase rate of the solid solubility of Nb in austenite calculated by extraction phase analysis was lower than that calculated by Ohtani’s equation. This may be attributed to Ohtani’s equation being based on the Fe-Nb-C ternary system, while the present study was based on the actual gear steels with different carbon contents. The alloying elements in the experimental steels, such as Cr, Ni, and Mo, could reduce the activity of carbon in austenite [19,20], which would reduce the amount of dissolution of NbC precipitates to some extent.

**Figure 3:**
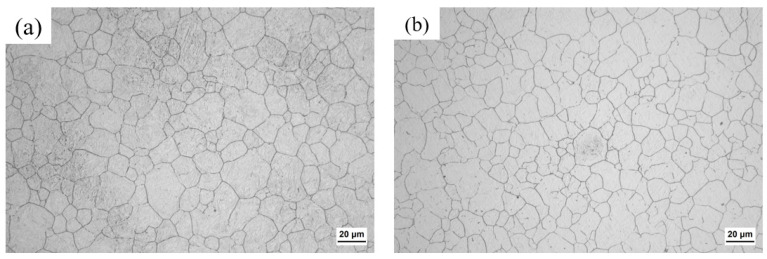
Optical micrographs showing prior austenite grains: (**a**) the case and (**b**) the core of the 930 °C carburized specimen.

Figure 3 shows the prior austenite grain sizes of the near-surface carburized layer and core of the specimen carburized at the temperature of 930 °C. The average prior austenite grain sizes of the near-surface carburized layer and core of the specimens were 12.13 μm and 11.39 μm, respectively, and the average prior austenite grain sizes of the near-surface carburized layer and core of the specimens at 980 °C were measured in the previous work, which was 15.17 μm and 15.07 μm, respectively [17]. It can be seen that the average prior austenite grain sizes of the near-surface carburized layer of the specimens were similar to those of the core of the specimens, and the average prior austenite grain sizes of the specimens had no remarkable difference at different carburized temperatures.

**Figure 4:**
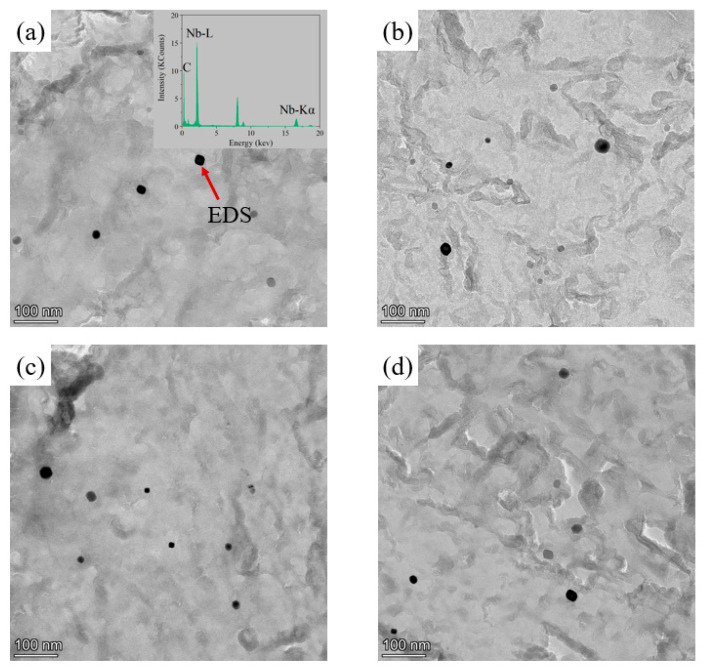
TEM micrographs showing NbC precipitates in (**a**) the case and (**b**) the core of the specimens carburized at 930 °C, and (**c**) the case and (**d**) core of the specimens carburized at 980 °C.

The NbC precipitates in the low-pressure carburized layer and core at different carburized temperatures were extracted by the carbon film, and the results are shown in Figure 4. The result of EDS showed that the black spherical precipitates were NbC precipitates as shown in Figure 4a. The average sizes of the precipitates in the carburized layer and core of the specimens carburized at 930 °C were 16.78 nm and 18.36 nm, respectively, and those of the specimens carburized at 980 °C were 21.79 nm and 22.63 nm, respectively. It can be seen that the average sizes of the precipitates in the carburized layer and core of the specimens carburized at different temperatures were about the same.

According to the Zener model, the growth rate of the grain diameter *D* is dependent on the competition between the grain growth’s driving force $P_{d}$ and the precipitate’s pinning pressure $P_{z}$, and the equation is expressed as [21],

(3) $$\frac{dD}{dt}=\left\{\begin{array}{c} M_{0}\exp\left(\frac{Q_{g}}{RT}\right)\left(P_{d}-P_{z}\right)\;\;\;\;\;\;\;\;\mathrm{if}\;\;\;\;\;\;\;P_{d}>P_{z}\\ 0\;\;\;\;\;\;\;\;\;\;\;\;\;\;\;\;\;\;\;\;\;\;\;\;\;\;\;\;\;\;\;\;\;\;\;\;\;\;\;\;\;\;\;\;\;\;\;\mathrm{if}\;\;\;\;\;\;\;P_{d}<P_{z} \end{array}\right.$$

where $M_{0}$ is a pre-exponential factor, R is the gas constant, and $Q_{g}$ is the activation energy of grain boundary mobility.

The driving pressure of the grain growth $P_{d}$ is expressed as [22],

(4) $$P_{d}=\alpha \frac{\gamma }{D}$$

where D is the average size of the grain, α is a coefficient with a value of 4 here [23], and γ is the interface energy.

The precipitate’s pinning pressure $P_{z}$ is calculated as follows [24,25]:

(5) $$P_{z}=\beta \gamma \frac{f}{<r>}$$

where β is a dimensionless constant assumed to be 12 [26], *f* is the precipitate’s volume fraction, which was measured by the extraction phase analysis method, and <r> is the average size of the precipitate, which was determined by the result of the carbon film.

When the $P_{d}$ is greater than the $P_{z}$, the austenite grains begin to grow. However, as the austenite grains grow, the $P_{d}$ decreases, which leads to a smaller growth rate of austenite. Until the $P_{d}$ is equal to the $P_{z}$, the austenite grain size $D_{lim}$ reaches the maximum at this time, and the $D_{lim}$ can be calculated as

(6) $$D_{lim}=\frac{\alpha <r>}{\beta f}$$

The calculated results of $D_{lim}$ of the low-pressure carburized layer and core at the carburizing temperature of 930 °C are shown in Table 4. It can be seen that the calculated results were about the same as the experimental results, which means that both the carburized layer and the core of the specimens were refined by grain boundaries pinning effect of NbC precipitates during the low-pressure carburizing process.

In summary, a much greater amount of the Nb element would be needed in the experimental steel to form enough NbC precipitates at high temperatures to maintain the refined grain size, according to Ohtani’s equation. In fact, both experimental and calculated results showed that adding 0.03 wt.% Nb to the steel could maintain the refined grain size during the high-temperature carburizing process. It is of great significance for the development of new gear steels suitable for the low-pressure carburizing process.

## 4. Conclusions

(1) The result of the extraction phase analysis method showed that experimental results were all smaller than the calculated result, and the difference between them was larger with the increase of carbon content. On the other hand, the equation for the variation of [Nb][C] solid solubility product with carbon content in gear steel was obtained:

$$\log\left\{\left[Nb\right]\left[C\right]\right\}=1.23-5020/T+\left[C\right]\left(1887/T-0.75\right)$$

(2) When the low-pressure carburized temperatures were 930 °C and 980 °C, the austenite grain sizes in the carburized layer and the core of the specimens had no remarkable difference;

(3) According to the result of the extraction phase analysis method and carbon film, both the carburized layer and the core of specimens were refined by pinning grain boundaries with NbC precipitates during the low-pressure carburizing process.

## Figures and Tables

**Table 1:** Chemical compositions of the experimental steels (wt.%).

Steels	C	Si	Mn	Cr	Ni	Mo	Nb
20C	0.21	0.02	0.77	1.59	1.56	0.31	0.040
60C	0.60	0.02	0.76	1.56	1.55	0.31	0.037
100C	0.99	0.02	0.75	1.60	1.51	0.32	0.040
140C	1.45	0.03	0.79	1.57	1.54	0.29	0.046
180C	1.82	0.03	0.81	1.61	1.55	0.32	0.052

**Table 2:** Extraction phase analysis results of experimental steels with different carbon contents (wt.%).

Steel	Temp. (°C)	Precipitate			In Austenite	
		Nb	C^※^	NbC	[Nb]	[C]
20C	930	0.030	0.0051	0.0351	0.010	0.1961
	980	0.028	0.0047	0.0327	0.012	0.1964
	1050	0.022	0.0036	0.0256	0.018	0.1972
	1100	0.011	0.0014	0.0124	0.029	0.1986
	1200	0.000	0.0000	0.0000	0.040	0.2000
60C	930	0.032	0.0056	0.0376	0.005	0.5984
	980	0.029	0.0051	0.0341	0.008	0.5989
	1050	0.028	0.0049	0.0329	0.009	0.5991
	1100	0.025	0.0040	0.0290	0.012	0.6000
	1200	0.012	0.0016	0.0136	0.025	0.6024
100C	930	0.032	0.0058	0.0378	0.008	0.9959
	980	0.031	0.0056	0.0366	0.009	0.9960
	1050	0.029	0.0053	0.0343	0.011	0.9963
	1100	0.024	0.0042	0.0282	0.016	0.9969
	1200	0.015	0.0026	0.0176	0.025	0.9981
140C	930	0.034	0.0061	0.0401	0.012	1.4449
	980	0.034	0.0060	0.0400	0.012	1.4450
	1050	0.030	0.0053	0.0353	0.016	1.4457
	1100	0.027	0.0047	0.0317	0.019	1.4463
	1200	0.020	0.0035	0.0235	0.026	1.4475
180C	930	0.032	0.0059	0.0379	0.020	1.8151
	980	0.029	0.0053	0.0343	0.023	1.8157
	1050	0.028	0.0050	0.0330	0.024	1.8160
	1100	0.026	0.0046	0.0306	0.026	1.8164
	1200	0.017	0.0029	0.0199	0.035	1.8181

(Note: C^※^ was calculated based on Nb content according to the stoichiometric ratio of NbC).

**Table 3:** The value of A and B of [Nb][C] solid solubility product equations in the experimental steels with different carbon contents.

Steel	A	B
20C	0.89	4361
60C	1.01	4246
100C	0.72	3439
140C	0.15	2348
180C	−0.23	1462

**Table 4:** The calculated result of $D_{lim}$ of the carburized layer and core at different carburized temperatures.

	Carburized Layer at 930 °C	Core at 930 °C	Carburized Layer at 980 °C	Core at 980 °C
α	4	4	4	4
β	12	12	12	12
<r>	16.78 nm	18.36 nm	21.79 nm	22.63 nm
f	0.000454	0.000420	0.000511	0.000499
Calculation	12.32 μm	14.57 μm	14.21 μm	15.12 μm
Experiment	12.76 μm	11.80 μm	15.17 μm	15.07 μm

## Data Availability

The original contributions presented in the study are included in the article, further inquiries can be directed to the corresponding author.
